# Malaria in rural Mozambique. Part II: children admitted to hospital

**DOI:** 10.1186/1475-2875-7-37

**Published:** 2008-02-26

**Authors:** Quique Bassat, Caterina Guinovart, Betuel Sigaúque, Pedro Aide, Jahit Sacarlal, Tacilta Nhampossa, Azucena Bardají, Ariel Nhacolo, Eusébio Macete, Inácio Mandomando, John J Aponte, Clara Menéndez, Pedro L Alonso

**Affiliations:** 1 Barcelona Center for International Health Research (CRESIB), Hospital Clínic/Institut d'Investigacions Biomèdiques August Pi i Sunyer, University of Barcelona, Rosselló 132, E-08036 Barcelona, Spain; 2 Centro de Investigação em Saúde de Manhiça (CISM), Manhiça, CP 1929, Maputo, Mozambique; 3 Instituto Nacional de Saúde, Ministério de Saúde, Maputo, Mozambique; 4 Faculdade de Medicina, Universidade Eduardo Mondlane, Maputo, Mozambique; 5 Direcção Nacional de Saúde, Ministério de Saúde, Maputo, Mozambique

## Abstract

**Background:**

Characterization of severe malaria cases on arrival to hospital may lead to early recognition and improved management. Minimum community based-incidence rates (MCBIRs) complement hospital data, describing the malaria burden in the community.

**Methods:**

A retrospective analysis of all admitted malaria cases to a Mozambican rural hospital between June 2003 and May 2005 was conducted. Prevalence and case fatality rates (CFR) for each sign and symptom were calculated. Logistic regression was used to identify variables which were independent risk factors for death. MCBIRs for malaria and severe malaria were calculated using data from the Demographic Surveillance System.

**Results:**

Almost half of the 8,311 patients admitted during the study period had malaria and 13,2% had severe malaria. Children under two years accounted for almost 60% of all malaria cases. CFR for malaria was 1.6% and for severe malaria 4.4%. Almost 19% of all paediatric hospital deaths were due to malaria. Prostration (55.0%), respiratory distress (41.1%) and severe anaemia (17.3%) were the most prevalent signs among severe malaria cases. Severe anaemia and inability to look for mother's breast were independent risk factors for death in infants younger than eight months. For children aged eight months to four years, the risk factors were malnutrition, hypoglycaemia, chest indrawing, inability to sit and a history of vomiting.

MCBIRs for severe malaria cases were highest in children aged six months to two years of age. MCBIRs for severe malaria per 1,000 child years at risk for the whole study period were 27 in infants, 23 in children aged 1 to <5 years and two in children aged ≥5 years.

**Conclusion:**

Malaria remains the number one cause of admission in this area of rural Mozambique, predominantly affecting young children, which are also at higher risk of dying. Measures envisaged to protect children during their first two years of life are likely to have a greater impact than at any other age.

## Background

Out of the 350–550 million malaria cases that are estimated to occur in the world every year [1,2], only around 1–2% are severe or life threatening [3-5]. However, this small proportion represents an enormous malaria death toll per year, especially in sub-Saharan Africa, where more than 90% of the malaria deaths are thought to take place every year, affecting mainly children and pregnant women [1,2,6]. Incidence rates of severe malaria among populations in endemic areas are difficult to estimate as the demographic information required is often unavailable, and morbidity data can often only be inferred from hospital records. Characterization of severe malaria syndromes among hospitalized African children has been previously done in different settings [7-12], and prognostic significance to the different clinical presentations has been attributed. Nevertheless, severe malaria features may change according to a number of factors including the genetic characteristics of the population, malaria epidemiology, health-seeking behaviour, non-malaria co-morbidity, clinical assessment and the local case management.

In Mozambique, as in other sub-Saharan African countries, malaria represents the main cause of paediatric outpatient consultations and admissions to hospital. However, no detailed characterization of the different malarial clinical syndromes on admission exists in the country. A comprehensive picture of the clinical and epidemiological characteristics of severe malaria is necessary to prioritize public health interventions and to guide national policies. This paper presents information on the clinical features, outcome and community incidences of malaria and severe malaria in children admitted to a rural hospital in Mozambique. Data on children with malaria who attend the outpatient clinic of the same hospital are presented in a companion article [13].

## Methods

### Study site and population

The study area is located in Manhiça, Maputo Province, southern Mozambique. The Manhiça Health Research Center (CISM) runs a Demographic Surveillance System in the area [14] and a morbidity surveillance system at Manhiça District Hospital. A detailed description of these and of the study area can be found in the companion article [13].

### Study design

Retrospective study of the data collected through the Manhiça morbidity surveillance system. This paper presents data from children younger than 15 years who were admitted to Manhiça District Hospital during a period of two years (1^st ^of June 2003 to 31^st ^of May 2005).

### Hospital surveillance system

A standardized admission questionnaire, which includes demographic, clinical and outcome data, was filled-in for all paediatric admissions (children less than 15 years of age) to the hospital. A physician or experienced medical officer performed a physical exam of the children on admission and completed the questionnaire. An open clinical process was also filled, where the daily clinical evolution was recorded during admission.

Laboratory data was also recorded on the admission questionnaire. Upon arrival a finger prick blood sample was collected into heparinized capillaries to measure packed cell volume (PCV) and blood glucose concentration, and thick and thin blood films were prepared to quantify *Plasmodium falciparum *parasitaemia.

HIV status information was not routinely collected. Admission criteria for children with malaria included any sign of severe disease (see definitions below), inability to take oral medication, or moderate anaemia with a risk of cardio-respiratory decompensation. Hyperparasitaemia, although mentioned as an admission criterion in the national guidelines, was rarely a cause of admission at Manhiça hospital unless accompanied by moderate anaemia or other severity symptoms.

Upon discharge or death up to four final diagnoses, based on the ICD classification of diseases, were recorded on the questionnaire by a project physician after review of clinical signs and symptoms and laboratory results.

### Case management

Children admitted with the diagnosis of malaria were managed according to Mozambican national guidelines, which, at the time of the study, included parenteral treatment with quinine (with an initial loading dose of 20 mg/kg plus subsequent 10 mg/kg doses, three times a day) for a minimum of six doses if completed with treatment with sulphadoxine-pyrimethamine (SP), or 21 doses when used as monotherapy. Treatment was switched to oral as soon as the child was able to tolerate. Blood transfusions were restricted to children with a PCV < 12% or to children with higher PCVs but with clinical signs of decompensation (respiratory distress or signs of heart failure) or neurological impairment. Hypoglycaemia was handled with intra-venous 30% dextrose solution, repeated as necessary, and convulsions treated with up to two doses of rectal or intra-venous diazepam, followed by intramuscular or intravenous phenobarbitone if seizures could not be controlled. Lumbar punctures were normally performed in children with a witnessed convulsion or a history of convulsions in the previous 24 hours, with positive meningeal signs, focal neurological signs, suspected clinical sepsis or a Blantyre Coma Score <5. Facilities for intensive care do not exist. All clinical assistance and treatment of admitted children is free of charge. Children requiring specialized care were transferred to Maputo Central Hospital.

### Laboratory methods

PCV was measured using a microcentrifuge and a Hawksley haematocrit reader card (Hawksley & Sons Ltd, Lancing, UK). Thick and thin blood films were air-dried, Giemsa-stained, and examined using a light microscope fitted with a 100× oil immersion lens. Slides were declared negative only after 2000 leukocytes have been counted. Parasite numbers were converted to a count/μL by assuming a standard leukocyte count of 8000/μL. Glycaemia was determined using Accu-Chek^® ^(Roche Inc., Manheim, Germany) at the bedside. All cerebrospinal fluids obtained from lumbar punctures were Gram-stained and cultured.

### Definitions

All case definitions were based on admission data from the admission questionnaires. An uncomplicated malaria case was defined as a child admitted with a clinical diagnosis of malaria with a *P. falciparum *asexual parasitaemia > 0 parasites/μL and not fulfilling the criteria for severe malaria.

Severe malaria was defined as a malaria case with at least one of the following criteria: PCV < 15%, deep coma (Blantyre coma score ≤ 2), prostration (inability to sit unaided or to look for mother's breast/feed in children who cannot yet sit), hypoglycaemia (< 2.2 mmol/L), convulsions (≥ 2 reported episodes in the 24 hours prior to admission) or respiratory distress (deep breathing or indrawing). Patients who met this definition but had a diagnosis of meningitis were excluded.

Impaired consciousness was defined as a child having a Blantyre coma score of less than 5. Severe anaemia was defined as a PCV < 15% on admission and cerebral malaria was defined as children with malaria and deep coma.

An increased respiratory rate (tachypnoea) was defined according to age: respiratory rate ≥ 60 breaths/min for children < 2 months; ≥ 50 for children 2 to < 12 months and ≥ 40 for children 1–5 years. Weight-for-age Z scores (WAZ) were calculated using the LMS method and the 2000 CDC Growth Reference.

The clinical processes of all children who had been classified as a malaria death based on the admission questionnaire were reviewed by a paediatrician and reclassified according to the clinical evolution and other concomitant diagnoses. A malaria death was defined as a death in an admitted child in which malaria was considered the only or main cause of death. Children with malaria parasitaemia for whom the cause of death was not malaria, were not considered severe malaria cases.

### Data management and statistical methods

All admission questionnaires were double entered using a programme written in FoxPro version 5.0 (Microsoft Corp., Seattle, WA, USA). Statistical analyses were done with Stata 9.0 (Stata Corp., College Station, TX, USA).

Minimum community-based incidence rates (MCBIRs) for malaria and severe malaria were calculated referring cases to population denominators establishing time at risk (child years at risk (CYAR)) inferred from the DSS census information.

Children did not contribute to the numerator or denominator for a period of 28 days after each episode of malaria or when they were outside the study area.

*Plasmodium falciparum *parasitaemia results were missing in 10.7% of admitted children and PCV in 9.8%. Almost half of the parasitaemia and PCV missing results were in children younger than one month or older than five years. Children with missing PCV or parasitaemia results had a significantly higher case fatality rate compared to children with non-missing results. Outcome was missing in 0.1% of children. Children with missing age were dropped from the analysis.

Case fatality rates (CFRs) were calculated for individual signs and symptoms and combinations of these as the number of patients who died having that clinical presentation divided by the total number of patients with known outcome admitted with that clinical presentation. These CFRs represent in-hospital mortality and do not include patients who absconded or were transferred.

The seasonality of malaria and severe malaria admissions was assessed, using November to April as the defined rainy season.

Qualitative variables were compared using a χ^2 ^test or Fisher's exact test. Means of normally distributed variables were compared using the Student's t-test or ANOVA. Geometric means of parasitaemia were compared using the Wilcoxon Rank sum test.

A multivariate regression analysis was done to assess risk factors for malaria death in children aged one month to five years with malaria, the age group that concentrates most malaria deaths. The analysis was done separately for children aged one to seven months and for children aged eight months to five years, given that the ability to locate a painful stimulus, strongly associated with the risk of death [9], is unreliable in the younger age group. Given that the dependent variable was the final outcome (dead/alive), only children with a known outcome were included in the analysis (those that absconded or were transferred and had no final outcome were excluded). A multivariate logistic regression was performed with malaria death or survival as the outcome, using an automated backward and forward stepwise estimation. All variables that were associated with death at a significance level of p < 0.10 in the univariate analysis were included in the initial model. The significance level for removal from the model was set at p = 0.06 and that for addition to the model at p = 0.05.

Thus, three different subgroups of data were used for the different analyses. The analysis of the **clinical presentation and case fatality rates of children admitted with malaria **includes all children who were admitted to the hospital during the study period (n = 8311). However, CFRs refer only to children with a known outcome. The multiple logistic regression analysis of the **risk factors for death in children admitted with malaria **was done for all children who were admitted with malaria during the study period and for which the final outcome (dead/alive) was known (n = 3859). Finally, the analysis of the **minimum community-based incidence rates **was restricted to study area children who were admitted with malaria (n = 1678).

## Results

### Clinical presentation and case fatality rates of children with malaria admitted to the hospital

During the two year study period, a total of 8,311 children younger than 15 years were admitted to Manhiça District Hospital. Almost half of them (4,080 – 49.1%) had a diagnosis of malaria, and for 2.8% the diagnosis was missing. 27.0% of all admitted malaria cases fulfilled the definition of severe malaria, which corresponds to 1,100 (13.2%) of all admitted patients.

Males accounted for 52.1% of the malaria (2,126/4,078, p = 0.007) and 53.5% of the severe malaria admissions (589/1,100, p = 0.02).

**Figure 1:**
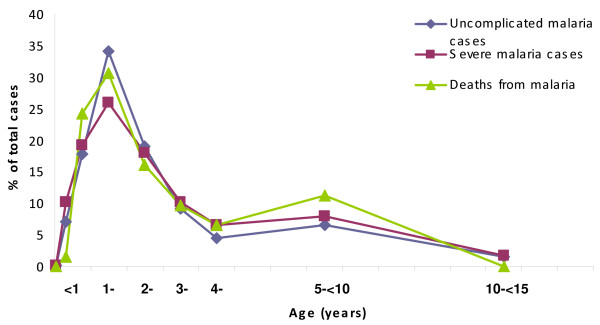
Age distribution of uncomplicated malaria, severe malaria and malaria deaths in children admitted to hospital.

**Figure 2:**
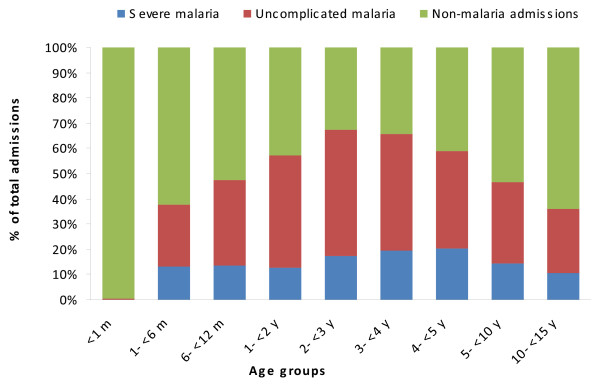
Relative contribution of uncomplicated malaria and severe malaria to hospital admissions according to age group.

The age distribution of uncomplicated malaria cases, severe malaria cases and malaria deaths among children admitted to the hospital is shown in Figure 1. The highest burden of malaria cases occurred among children younger than five years of age (91.4%), with more than half (58.1%) of the cases occurring in the first two years of life. Infants carried the brunt of severe malaria, accounting for almost 30% of the cases. Malaria was very rare in the neonatal period, with only a few cases diagnosed, but was fairly frequent in the first six months of life. Figure 2 shows the relative contribution of malaria (severe and uncomplicated) to hospital admissions in children younger than 15 years of age at Manhiça District Hospital, according to age group.

The CFR for all admitted children was 4.0% (332/8,311). During the study period, there were 62 deaths attributable to malaria, representing 18.7% of all paediatric in-hospital deaths. The age distribution of malaria deaths was similar to that of malaria cases (Figure 1), although very few of those deaths occurred in the first six months of life. While the majority of malaria deaths were clustered in the first two years of life, one in ten deaths occurred in children five to ten years of age. The case fatality rate for malaria (1.6%) was significantly lower than that for other diagnoses (7.3%, p < 0,0001). The CFR for severe malaria cases was 4.4%. There were no statistically significant differences in malaria CFRs by age group (data not shown).

Table 1 summarizes some key characteristics among malaria admissions. Although nearly all children had a history of fever, one third of all malaria cases requiring admission did not have fever on arrival. The median of reported fever days prior to admission was of two days, with no significant differences between uncomplicated malaria and severe malaria cases or between non-fatal and fatal malaria cases. The median length of stay in hospital for malaria cases was three days, but among fatal cases, more than 50% died within the first 48 hours.

**Table 1:** Anaemia, fever, parasitaemia and duration of admission among hospitalized malaria cases

	**Total malaria cases**	**Uncomplicated malaria cases**	**Severe malaria cases**	**Signif.**^#^
1. PCV* on admission in children with malaria	**% (n/N)**	**% (n/N)**	**% (n/N)**	
25–< 33%	32.3% (1302/4027)	33.5% (985/2945)	29.3% (317/1082)	p = 0.01^&^
15–< 25%	48% (1931/4027)	52.0% (1532/2945)	36.9% (399/1082)	p < 0.0001^&^
< 15%	4.6% (187/4027)	0.0% (0/2945)	17.3% (187/1082)	
Mean (SD) PCV	24.7% (6,9)	25.2% (6,3)	23.4% (8,3)	p < 0.0001^§^
**2. Fever (on admission)**	**% (n/N)**	**% (n/N)**	**% (n/N)**	
History of fever in the past 24 h	99.4% (4056/4079)	99.5% (2965/2979)	99.2% (1091/1100)	p = 0.19^&^
Axillary temperature ≥ 37,5°C	66.1% (2692/4074)	65.0% (1933/2975)	69.1% (759/1099)	p = 0.01^&^
Axillary temperature ≥ 39°C	34.7% (1413/4074)	35.1% (1045/2975)	33.5% (368/1099)	p = 0.33^&^
Median (IQR^$^) length of reported fever before admission (days)	2 (1;3)	2 (1;3)	2 (1;3)	
**3. Parasitaemia on admission**				
Geometric mean parasitaemia (95% CI) (parasites/μL)	18838.3 (17732.6–20013.0)	20615.8 (19250.0–22078.5)	14755.7 (13022.5–16719.5)	p < 0.0001^##^
**4. Duration of admission**				
Median (IQR^$^) length of stay (days)	3 (2;4)	3 (2;4)	3 (2;4)	

*PCV: Packed Cell Volume

^# ^Significance: p-value of the statistical test comparing uncomplicated with severe cases

^&^χ^2 ^test

§t-test

$ Inter quartile range

^## ^Wilcoxon rank sum test

Mean PCVs among malaria patients significantly increased with increasing age (data not shown) and were significantly lower for severe malaria cases (23.4%, SD 8.3%) than for uncomplicated cases (25.2%, SD 6.3%; p < 0.0001). Severe malaria cases had a significantly lower geometric mean parasitaemia (14,755.7 parasites/μL) than non-severe malaria cases (20,615.8 parasites/μL, p < 0.0001). The geometric mean parasitaemias among malaria deaths was also similarly lower when compared to non-lethal cases. Case fatality rates by parasitaemia level are shown in Table 2. Overall, case fatality rates tended to be higher for lower parasitaemias, although differences were not significant.

**Table 2:** Prevalence and associated case fatality rates of different signs on admission in malaria patients

	**Prevalence among total malaria patients**		**CFR in total malaria patients**		**Prevalence among severe malaria patients**		**CFR in severe malaria patients**	
	
	**% (n/N)**	**95% CI**	**% (n/N)**	**95% CI**	**% (n/N)**	**95% CI**	**% (n/N)**	**95% CI**
**Commonly used criteria for severe malaria**								
Severe Anaemia (PCV < 15%)	4.6% (187/4027)	4.0–5.3	5.7% (9/159)	2.6–10.5	17.3% (187/1082)	15.1–19.7	5.7% (9/159)	2.6–10.5
Deep Coma (BCS ≤ 2)	0.6% (24/4078)	0.4–0.9	18.2% (4/22)	5.2–40.3	2.2% (24/1099)	1.4–3.2	18.2% (4/22)	5.2–40.3
Repeated (≥2/24 h) convulsions	2.4% (98/4076)	2.0–2.9	4.4% (4/91)	1.2–10.9	8.9% (98/1099)	7.3–10.8	4.4% (4/91)	1.2–10.9
Hypoglycaemia (<2.2 mmol/L)	1.0% (40/3904)	0.7–1.4	16.2% (6/37)	6.2–32.0	3.7% (39/1046)	2.7–5.1	16.2% (6/37)	6.2–32.0
Prostration	14.9% (607/4080)	13.8–16.0	5.1% (29/564)	3.5–7.3	55.0% (605/1100)	52.0–58.0	5.2% (29/563)	3.5–7.3
Respiratory distress	11.1% (454/4076)	10.2–12.1	6.1% (25/407)	4.0–8.9	41.1% (452/1100)	38.2–44.1	6.2% (25/405)	4.0–9.0
**Other Signs**								
Impaired consciousness (BCS < 5)	2.1% (86/4078)	1.7–2.6	10.0% (8/80)	4.4–18.8	6.5% (71/1099)	5.1–8.1	12.3% (8/65)	5.5–22.8
Jaundice	1.2% (47/4076)	0.8–1.5	0% (0/43)	-	1.9% (21/1100)	1.2–2.9	0% (0/20)	-
Transfusion recipients	14.4% (586/4065)	13.3–15.5	2.8% (16/568)	1.6–4.5	29.1% (320/1099)	26.4–31.9	4.5% (14/310)	2.5–7.5
Dehydration (mod./sev.)	3.4% (139/4076)	2.9–4.0	6.2% (8/129)	2.7–11.9	5.8% (64/1100)	4.5–7.4	10.3% (6/58)	3.9–21.2
Number of severity criteria*								
Only one					78.4% (808/1031)	75.7–80.8	3.0% (23/762)	1.9–4.5
Two					16.6% (171/1031)	14.4–19.0	8.3% (12/145)	4.3–14.0
Three					4.2% (43/1031)	3.0–5.6	13.2% (5/38)	4.4–28.1
Four or more					0.9% (9/1031)	0.4–1.7	42.9% (3/7)	9.9–81.6
**Parasitaemia on admission (parasites/μL)**								
1 to 4,999	20.8% (848/4080)	19.5–22.1	2.2% (17/788)	1.3–3.4	25.7% (283/1100)	23.2–28.4	4.2% (11/259)	2.1–7.5
5,000 to 19,999	17.5% (713/4080)	16.3–18.7	2.0% (13/666)	1.0–3.3	17.7% (195/1100)	15.5–20.1	6.2% (11/177)	3.1–10.8
20,000 to 99,999	43.7% (1783/4080)	42.2–45.2	1.3% (22/1695)	0.8–2.0	40.3% (443/1100)	37.4–43.2	3.9% (16/411)	2.2–6.2
≥ 100,000	18.0% (736/4080)	16.9–19.3	1.4% (10/710)	0.7–2.6	16.3% (179/1100)	14.1–18.6	4.1% (7/169)	1.7–8.3

* Any of the following: Severe anaemia, deep coma, repeated convulsions, hypoglycaemia, prostration or respiratory distress

**Figure 3:**
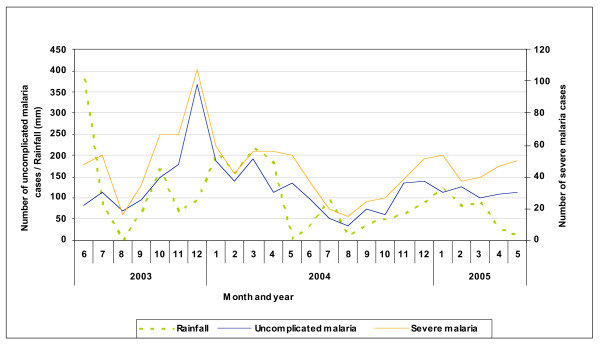
Seasonality of cases of malaria (severe and uncomplicated) admitted to the hospital and rainfall pattern.

62.8% of the malaria and 59.5% of the severe malaria cases (p < 0.0001), 59.4% of severe malarial anaemia episodes (p = 0.006) and 64.5% of deaths attributable to malaria occurred during the rainy season (p = 0.03) (Figure 3). No clear-cut seasonal pattern was found for the different syndromic presentations.

### Characterization of severe malaria cases

Table 2 shows the prevalence and case fatality rates of the commonly used criteria for defining severe malaria, as well as of other signs and symptoms. Among severe cases, prostration was the most frequently observed severe malaria criterion (55.0% of total severe malaria cases), followed by respiratory distress (41.1%) and severe anaemia (17.3%). Deep coma (BCS ≤ 2), which was infrequent, had the highest associated CFR (18.2%), followed by hypoglycaemia (16.2%) and respiratory distress (6.2%). Among other signs and symptoms, impaired consciousness (BCS < 5) and moderate to severe dehydration had CFRs of 12.3% and 10.3% respectively. These CFRs were lower when considering total malaria patients.

**Figure 4:**
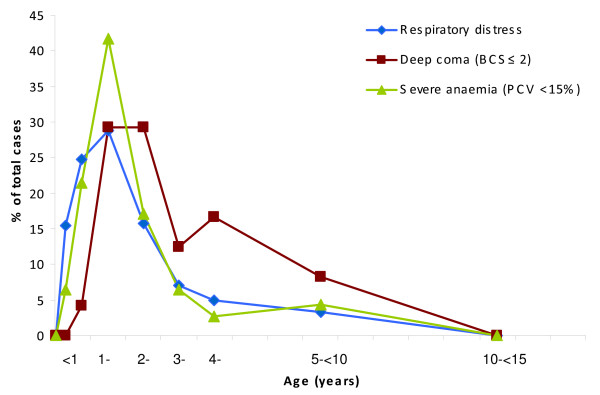
Age distribution of severe malaria cases according to syndromic presentation.

Geometric mean parasitaemias in malaria cases on admission were higher for deep coma patients (26,241.5 parasites/μL) than for severely anaemic children (11,924.1 parasites/μL) or children with respiratory distress (11,425.6 parasites/μL). Figure 4 represents the age distribution of these three classical syndromic presentations of severe malaria.

78.4% of children with severe malaria presented on admission with a single severe malaria criterion, 16.6% had two criteria and only 5.1% had three or more criteria. The case fatality rates significantly increased with increasing number of criteria fulfilled (p < 0.0001; Table 2).

### Risk factors for death in children admitted with malaria

During the study period there were 3,859 children admitted with malaria and with complete survival data, of which 62 died (CFR of 1.6%). None of these deaths occurred in the neonatal period and only seven in children above five years of age. The analysis of risk factors for death in malaria cases was restricted to children aged one month to five years (55 deaths), the age group where most deaths occur. Results are presented separately for children aged one to seven months and eight months to five years. Additional file 1 shows by age group the prevalence and CFRs of signs and symptoms significantly associated with death in the univariate analysis in children admitted with malaria. Table 3 presents the risk factors independently associated with death in children with malaria admitted to the hospital. In children aged one to seven months, severe anaemia and inability to look for mother's breast were independently associated with an increased risk of death. In children aged eight months to five years two logistic regression models were obtained. Hypoglycaemia, inability to sit, malnutrition (WAZ) and history of vomiting were clear independent risk factors for death in both models. There was collinearity between nasal flaring and indrawing. The model with indrawing had a better fit according to the Akaike's information criterion (AIC) than the model with nasal flaring and is thus the one presented.

**Table 3:** Risk factors independently related to mortality in children < 5 years with malaria, according to a multivariate logistic regression model

Risk factor	Adjusted OR	95% CI	p (LR X^2 ^test 1 d.f.)
**Age 1–7 months (n = 524)**			
Severe anaemia (PCV < 15%)	9.5	2.0–45.5	0.01
Inability to look for mother's breast	4.6	1.1–19.1	0.04
			
**Age 8 months-<5 years (n = 2667)**			
Hypoglycaemia	8.2	2.2–31.0	0.005
Indrawing	5.8	2.8–12.1	<0.0001
Weight-for-age z-score (per unit increase)	0.66	0.53–0.82	0.0003
Inability to sit	4.5	2.1–9.3	0.0001
History of vomiting	2.4	1.1–5.0	0.03

### Minimum community-based incidence rates

The age-specific minimum community-based incidence rates (MCBIRs) for malaria and severe malaria in the study area are shown in Table 4. Malaria incidence in the neonatal period was negligible, but then increased rapidly with age and children from six to 24 months of age had the highest MCBIRs for malaria and severe malaria. The number of cases does not coincide with the numbers given above, as only study area children were included to calculate the MCBIRs.

**Table 4:** Minimum community-based incidence rates of admitted malaria (total and severe) cases per 1,000 child years at risk, by year of study

		**1st June 2003–31th may 2004**			**1st June 2004–31th may 2005**		
	**Age group**	**N° of cases/CYAR**	**Rate per 1000 CYAR**	**95% CI**	**N° of cases/CYAR**	**Rate per 1000 CYAR**	**95% CI**

**Total malaria cases**	<1 month	0/155	0	-	1/153	6.5	0.9–46.4
	1–<6 m	65/800	81.2	63.7–103.6	14/764	18.1	10.7–30.5
	6–<12 m	184/889	207.0	179.2–239.2	66/890	74.1	58.2–94.4
	1–<2 y	304/1626	187.0	167.1–209.2	169/1731	97.6	84.0–113.5
	2–<3 y	199/1486	133.9	116.6–153.9	134/1605	83.5	70.5–98.9
	3–<4 y	98/1504	65.2	53.5–79.4	67/1494	44.8	35.3–57.0
	4–<5 y	70/1499	46.7	36.9–59.0	34/1521	22.3	16.0–31.3
	5–<10 y	80/6812	11.7	9.4–14.6	51/7100	7.2	5.5–9.5
	10–<15 y	12/5342	2.2	1.3–4.0	12/5666	2.1	1.2–3.7
							
**Severe malaria cases**	<1 month	0/155	0	-	0/153	0	-
	1–<6 m	22/803	27.4	18.0–41.6	6/774	7.7	3.5–17.2
	6–<12 m	51/898	56.8	43.1–74.7	19/894	21.3	13.6–33.3
	1–<2 y	60/1644	36.5	28.3–47.0	42/1741	24.1	17.8–32.6
	2–<3 y	55/1497	36.8	28.2–47.9	30/1614	18.6	13.0–26.6
	3–<4 y	30/1509	19.9	14.9–28.4	22/1498	14.7	9.7–22.3
	4–<5 y	32/1502	21.3	15.1–30.1	13/1523	8.5	5.0–14.7
	5–<10 y	27/6816	4.0	2.7–5.8	19/7102	2.7	1.7–4.2
	10–<15 y	2/5343	0.4	0.1–1.5	6/5667	1.1	0.5–2.4

CYAR: child years at risk

95% CI: 95% confidence interval

MCBIRs for the total paediatric population admitted to the hospital were of 50 and 26 episodes per 1,000 CYAR for clinical malaria and 14 and seven cases per 1,000 CYAR for severe malaria in the first and second study year respectively. MCBIRs for clinical malaria per 1,000 child years at risk for the whole study period were 90 in infants, 86 in children aged 1 to <5 years and 6 in children aged ≥ 5 years, whereas for severe malaria, the numbers were 27 in infants, 23 in children aged 1 to <5 years and two in children aged ≥5 years.

The overall MCBIRs for malaria and severe malaria, as detected through hospital passive case detection, were twice as high during the first year of study than during the second year and the age patterns were different. In the first year, incidences were highest in infants and decreased with age, whereas in the second year the burden was slightly shifted to the right and children aged one to four years carried the highest burden of malaria and severe malaria.

## Discussion

This is a retrospective study of paediatric malaria cases admitted to a rural hospital in southern Mozambique. The sex and age-pattern of admitted children was similar to that reported in other countries in the region [9,15]. Data for very young infants and children between 5 and 15 years of age, seldom available in the malaria literature, are also presented in this paper. Males accounted for a significantly higher number of admissions due to malaria and severe malaria, but also for admissions other than malaria, reflecting either a local gender-bias in treatment-seeking behaviour, exposure or an increased susceptibility to severe disease. Uncomplicated malaria and severe malaria cases and deaths attributable to malaria decreased with age in the first five years of life. Indeed, more than 50% of all admitted malaria cases, including life-threatening and fatal ones, occurred in the first two years of life. This confirms the importance of malaria among children ≤ 2 years of age in this area, despite being considered mesoendemic on the basis of the entomological data (estimated entomological inoculation rate of 38 infective bites per person per year [16]). The proportion of severe cases among infants and young children is not very different from that found in a high malaria transmission area in Tanzania [9], suggesting that the burden of malaria morbidity and mortality is borne mainly by infants and young children and this may be relatively independent of the intensity of transmission. This also suggests the potential significant impact in reducing the burden of malaria by malaria control interventions targeted at early infancy, such as intermittent preventive treatment in infants (IPTi) [17], and reinforces the need to implement malaria control measures specifically for this age group. Moreover, the real contribution of malaria may possibly be underestimated in both the neonatal period and the oldest children, as half of the missing slide data corresponded to these two age groups, and efforts should be made in the future to strengthen their slide collection.

The age distribution and prevalence of the three classical severe malaria clinical syndromes (respiratory distress, severe anaemia and coma) is also concordant with a pattern of moderate transmission [18]. Respiratory distress and anaemia, together with the clinical sign prostration, were the most prevalent presentations of severe malaria, with the majority of cases occurring in younger children, as opposed to coma, infrequent and slightly shifted to older children. Nevertheless, this should be interpreted with caution, as the assessment of neurological status in children younger than 8 months, and therefore the percentage of those with low Blantyre coma scores, is unreliable in this age group [9,19].

The case fatality rate of hospitalized malaria cases in this study (1.6%) was slightly lower than those recently published for six other African sites in the largest severe malaria study performed so far in Africa (ranging from 2.0 to 9.7%) [20]. This may reflect either a lower admission threshold for malaria patients in this site, implying a potential over-admission of patients, or different accessibility to hospital, leading to children arriving in less severe conditions. Alternatively, free access to health services in the country may also explain a wider and earlier use of health facilities.

Sixty-two malaria-attributable deaths occurred during the two year study period. Among children who died with malaria, a quarter died the same day of admission and more than half within the first 48 h of arriving to hospital. Most of those cases (73%) were already characterized as severe on admission, and the rest (27%) worsened during their stay in hospital before actually dying. Most of these early deaths occurred in children arriving to hospital in a much evolved and often irreversible clinical condition, raising the concern of how rapid access to hospital may determine the prognosis. In such cases, only aggressive resuscitation interventions, including artificial ventilation, may have had an impact on the outcome, but such measures are unavailable in most African settings. Early recognition of severe malaria cases at home or at the community level [21] and prompt pre-referral treatment, with effective and easy to administer antimalarials, such as rectal artesunate [22], may improve their survival likelihood.

Little has been published concerning severe malaria in Mozambique [23,24]. The results show the association between several characteristics on admission and a high risk of dying or a higher case fatality rate. However, caution should be taken when interpreting these type of data and it is essential to distinguish between the likelihood of a fatal outcome in a patient with certain symptomatology and the real contribution of malaria to such death. Hypoglycaemia and respiratory distress (including whichever presentation) have already been associated in previous studies in other African countries with a higher risk of death in malaria cases [7-10,12,23,25]. These results corroborate their importance, as they were found to be independent risk factors for death and presented high associated case fatality rates.

Alongside malaria, malnutrition is a recurrent underlying problem in all major child mortality causes [12,26], and this is reflected by the higher risk of death as the weight-for-age z-score decreases, also seen in other studies [12,27]. Co-infection with HIV/AIDS, not analysed in this study, may also play an important role in the outcome of malaria illness or in the vulnerability to develop a more severe presentation of the disease [28,29]. The magnitude of the overlapping malnutrition-immunosuppression syndrome, and the relevance of malaria infection in the progression of HIV infection towards AIDS in children, needs also to be specifically assessed.

Neither deep coma nor impaired consciousness were associated in this model with a higher risk of dying, contrary to what most studies have shown [7-10,12,25,27,30,31]. However, the low prevalence of those two conditions (0.6% and 2.1% respectively) found among malaria patients in Manhiça, when compared to other sites [20], suggests that this may reflect a problem of low numbers rather than of a true lack of significance. Despite being infrequent, coma still carries the highest case fatality rate.

The identification of the real cause of the respiratory distress in severe malaria patients remains unclear. Neither peripheral lactate determinations, nor acid/base statuses were performed during the two years of the study, and acidosis was only diagnosed on clinical grounds. The real importance of metabolic acidosis in this setting, as well as additional assessment of the incidence of overlapping respiratory infections (viral, bacterial or parasitic) need to be further investigated.

Prostration (as defined above), present in more than half of the severe malaria cases, was also identified as a major risk factor for death in both age groups. Identifying prostrated children at the outpatient clinic may be challenging, as it may be difficult to assess and depends on age, but it is likely to have a great impact on their survival.

A history of vomiting was also an independent risk factor for death, probably indicating an underlying dehydration, as vomiting often leads to clinical dehydration, implying an urgent need for parenteral treatment. In Tanzanian children [9] dehydration was associated with a higher risk of death, possibly reflecting the same mechanism. The hypothetical volume depletion among severe malaria patients, and the need for urgent and vigorous re-hydration measures, has become in recent years a subject of discussion [32,33]. Although these results can in no way shed light on this topic (due to lack of analytical data, electrolytes and subjectivity of the clinical diagnosis), the importance of rapidly diagnosing and treating clinical dehydration in such patients needs to be stressed, as it may improve their likelihood of surviving.

Anaemia, from mild to life-threatening, is highly prevalent among severe malaria patients. The real contribution of malaria to the burden of anaemia, the role of iron and other micronutrient deficiencies in anaemia, as well as the prevalence of haemoglobinopathies, still remains unclear in the Manhiça area. Preliminary data show that haemoglobinopathies, especially sickle cell disease, are relatively uncommon as compared to other African countries (Menéndez C, personal communication) and the effect on the risk of severe malaria of this low prevalence of a traditionally considered "protective" genetic trait, such as haemoglobin AS, is uncertain.

Despite the existence of a life-saving intervention for severe anaemia, this highly prevalent presentation of severe malaria is the most important risk factor for death (adjusted OR 9.5) in children younger than eight months. Interventions focused on this age group, and targeted at preventing anaemia could be highly beneficial.

These data show that the level of parasitaemia is not directly related to a higher risk of death. On the contrary, children with lower parasitaemias showed a tendency to have higher CFRs than children with higher parasite numbers, although differences were not significant. Parasitaemia level was not a significant risk factor in either the univariate or multivariate analysis. Poor outcome associated with low peripheral parasitaemias, has been previously associated to sequestration in the microvasculature [9,34], but among the deep coma cases in this setting, traditionally considered to suffer sequestration, higher geometric mean parasitaemias were found when compared with other malaria syndromes, such as anaemia or respiratory distress. Correlation between severity of disease and peripheral parasitaemia is not clear-cut and the latter may only play a small role in the overall complexity of severe malaria syndromes [4,35].

Fever on admission was absent in a third of the severe malaria patients, but a referred history of fever was almost omnipresent. Relying on the presence of fever or on the parasite count for identifying severe cases on admission may, therefore, be dangerous. Considering the high mortality associated to the first hours of admission, it would seem advisable that all ill children hospitalized with malaria parasitaemia receive prompt parenteral treatment, regardless of the parasitaemia level.

This paper describes MCBIRs for severe malaria ranging from seven to 14 cases per 1,000 CYAR for the whole paediatric population, but rising up to 17–29 cases per 1,000 CYAR in children between one and five years of age. These findings are concordant with MCBIRs found in children of similar ages in other settings [15,36]. These MCBIR findings are slightly lower than the 38 episodes per CYAR that can be inferred from a cohort of around 1,000 children (aged one to four years at enrolment) participating in the control group of a randomized controlled trial of the RTS,S malaria vaccine candidate within the same time period in the Manhiça study area [16,37]. The malaria vaccine study participants were closely followed-up for 18 months, and offered transport whenever they were found with fever, possibly leading to a higher rate of malaria (and severe malaria) diagnosis. Moreover, and as opposed to this series, where severe malaria was only diagnosed according to admission criteria, children in the malaria vaccine study were assessed throughout the whole hospitalization period, thus capturing severe malaria cases which may have not been classified as such on arrival.

Limitations arise when trying to estimate MCBIRs for both the neonates and the oldest children. Neonates are often admitted before their permanent identification number has been issued by the DSS, and the oldest children are more prone to present to hospital without reliable identification. Incidence rates can only be calculated among patients identified as belonging to the study area and may thus not include many malaria cases in those children. The substantial malaria burden among older children seen at the outpatient department [13], and the potential underestimation of the disease among the older admitted children suggests that malaria control programmes cannot disregard this specific age group.

MCBIRs for hospitalized malaria were in general twice as high during the first year of study follow-up. The incidence of outpatient malaria was also higher for all paediatric age groups during the first year [13]. Among hospitalized children with malaria, these differences were highest in infants, who were admitted with malaria or severe malaria three times less frequently in the second than in the first year of the study. These yearly differences could illustrate the high interannual variability that can occur within the same malaria endemic area or more likely reflect a true decline in the malaria burden possibly explained by a series of different coincident factors. The superior rainfall in the first 12 months of data collection (June 2003–May 2004), the introduction in June 2003 of a new more effective first-line antimalarial treatment (amodiaquine and sulphadoxine-pyrimethamine), and a better and more qualified triage and initial assessment of the children arriving to hospital, may all have played a key role in reducing malaria transmission. The concomitant progress of different intervention trials of malaria control tools (RTS,S malaria vaccine and intermittent preventive treatment in infants and pregnant women), targeted to this same paediatric population, and ongoing during the study period, were probably also influential.

Hospital-based results underestimate the real burden of disease and are greatly influenced by health-seeking behaviour. In a verbal autopsy study in the Manhiça DSS area, 53% of all paediatric deaths occurred at home (Sacarlal J, personal communication), thus cases seen at the hospital may only be a fraction of the total paediatric community deaths [38]. Nevertheless, hospital-based data are often the only available data and are useful indicators of the health status of a population.

## Conclusion

This paper reviewed all malaria admissions during two years in a rural hospital of Mozambique, in which malaria still represents the principal cause of admission and an important cause of in-hospital deaths in the paediatric ward. Measures targeted at encouraging families to promptly take their sick children to hospital, at recognizing the risk factors for severity at presentation, and improving the initial care and management during admission, would be helpful to reduce malaria-attributable deaths. In populations with difficult access to hospitals, adequate pre-referral effective antimalarials should otherwise made available.

## Authors' contributions

The hospital surveillance system and the DSS in Manhiça were designed by JJA, CM and PLA. They were set up in 1996 and have received numerous contributions to its design and implementation since then. During the study period QB, EM, PA, BS, AB, JS and TN were involved in the diagnosis and management of malaria patients and collection of data. AN coordinates the DSS in Manhiça. QB and CG led the analysis, interpretation and write up of this data set, and received input from all authors. All authors read and approved the final manuscript.

## Supplementary Material

Additional file 1 Prevalence and CFRs of signs and symptoms significantly associated with death in the univariate analysis in children admitted with malaria, according to age. Click here for file
