# Supplementary material for: Malaria in rural Mozambique. Part II: children admitted to hospital
Source: Malar J. 2008 Feb 26;7:37. doi: 10.1186/1475-2875-7-37 (PMC2275288; doi:10.1186/1475-2875-7-37)
Supplement: Additional file 1 — Prevalence and CFRs of signs and symptoms significantly associated with death in the univariate analysis in children admitted with malaria, according to age. [file 1475-2875-7-37-S1.doc]

**Prevalence of signs and symptoms and risk factors for death in children admitted with malaria**

|  | **Prevalence** | | **Mortality** | | | | |
| --- | --- | --- | --- | --- | --- | --- | --- |
| **Sign/Symptom^§^** | **n/N** | **%** | **N** | **% (CFR)** | **OR** | **95% CI** | **p-value** |
| **Children 1-7 months (n=557)** | | | | | | | |
| Skin fold | 28/556 | 5.0 | 2 | 7.1 | 4.0 | 0.8-19.2 | 0.06 |
| Pallor | 143/556 | 25.7 | 7 | 4.9 | 4.2 | 1.3-13.6 | 0.009 |
| Inability to look for mother’s breast | 89/534 | 16.7 | 7 | 7.9 | 9.4 | 2.6-33.6 | <0.0001 |
| PCV <15% | 19/550 | 3.6 | 3 | 15.8 | 10.9 | 2.6-45.1 | <0.0001 |
| Impaired consciousness | 87/557 | 15.6 | 4 | 4.6 | 2.8 | 0.8-9.5 | 0.09 |
| WAZ |  |  |  |  |  |  |  |
| >-1SD | 294/551 | 53.4 | 3 | 1 | 1 |  |  |
| -1 to -3 SD | 222/551 | 40.3 | 5 | 2.3 | 2.2 | 0.5-9.5 | 0.266 |
| <-3 SD | 35/551 | 6.4 | 2 | 5.7 | 5.9 | 0.9-37.0 | 0.03 |
| **Children 8 months-<5 years (n=2964)** | | | | | | | |
| History of dyspnea | 292/2955 | 9.9 | 18 | 6.2 | 6.9 | 3.7-12.9 | <0.0001 |
| Hist. of diarrhoea | 432/2962 | 14.6 | 11 | 2.6 | 2.0 | 1.0-4.1 | 0.04 |
| Hist. of vomiting | 579/2963 | 19.5 | 15 | 2.6 | 2.2 | 1.2-4.2 | 0.01 |
| Hist. of poor breast-feeding/stopped drinking | 118/2960 | 4.0 | 5 | 4.2 | 3.3 | 1.3-8.5 | 0.01 |
| Stopped eating | 194/2923 | 6.6 | 8 | 4.1 | 3.4 | 1.6-7.5 | 0.001 |
| Hist. of seizures | 243/2962 | 8.2 | 9 | 3.7 | 3.0 | 1.4-6.4 | 0.002 |
| Tachypnea^#^ | 1155/2951 | 39.1 | 28 | 2.4 | 3.4 | 1.8-6.6 | 0.0001 |
| Skin fold | 109/2960 | 3.7 | 9 | 8.3 | 7.7 | 3.6-16.6 | <0.0001 |
| Dehydration (moderate/severe) | 99/2961 | 3.3 | 7 | 7.1 | 6.0 | 2.6-13.8 | <0.0001 |
| Pallor | 692/2961 | 23.4 | 16 | 2.3 | 2.0 | 1.1-3.7 | 0.03 |
| Oedema | 94/2961 | 3.2 | 5 | 5.3 | 4.2 | 1.6-10.9 | 0.002 |
| Skin flaking off | 9/2961 | 0.3 | 1 | 11.1 | 8.7 | 1.1-71.0 | 0.02 |
| Oral candidiasis | 15/2961 | 0.5 | 2 | 13.3 | 10.9 | 2.4-50.1 | 0.0001 |
| Chest indrawing | 275/2961 | 9.3 | 20 | 7.3 | 9.1 | 4.9-16.9 | <0.0001 |
| Nasal flaring | 221/2961 | 7.5 | 18 | 8.1 | 9.6 | 5.1-18.1 | <0.0001 |
| Deep breathing | 39/2961 | 1.3 | 6 | 15.4 | 14.2 | 5.6-36.2 | <0.0001 |
| Crepitations/crackles | 174/2961 | 5.9 | 7 | 4.0 | 3.2 | 1.4-7.3 | 0.004 |
| Wheeze/roncus | 103/2961 | 3.5 | 4 | 3.9 | 2.9 | 1.0-8.3 | 0.04 |
| Gallop | 220/2961 | 7.4 | 7 | 3.2 | 2.5 | 1.1-5.6 | 0.03 |
| Splenomegaly | 1029/2961 | 34.8 | 8 | 0.8 | 0.4 | 0.2-0.9 | 0.03 |
| Impaired consciousness (BCS <5) | 64/2962 | 2.2 | 7 | 10.9 | 9.8 | 4.1-23.0 | <0.0001 |
| Inability to sit (≥ 6m) | 382/2915 | 13.1 | 19 | 5.0 | 6.7 | 3.5-12.5 | <0.0001 |
| Hypoglycaemia* | 28/2843 | 1.0 | 5 | 17.9 | 16.3 | 5.8-45.7 | <0.0001 |
| PCV <15% | 133/2929 | 4.5 | 6 | 4.5 | 3.6 | 1.5-8.8 | 0.002 |
| WAZ |  |  |  |  |  |  |  |
| >-1SD | 896/2922 | 30.7 | 5 | 0.6 | 1 |  |  |
| -1 to -3 SD | 1494/2922 | 51.1 | 15 | 1.0 | 1.8 | 0.7-5.0 | 0.25 |
| <-3 SD | 532/2922 | 18.2 | 21 | 4.0 | 7.3 | 2.7-19.7 | <0.0001 |

^#^ Tachypnea defined following WHO criteria: respiratory rate per minute >=60 in <2m, >=50 in 2-12m, >=40 in 1-5y.

* Hypoglycaemia defined as < 2.2 mmol/L

^§^ The following non-significant variables were also analysed: history of fever, history of cough, measured fever, tachycardia, jaundice, orange hair, lymph nodes, hepatomegaly.
